# Heart rate variability in horses with and without severe equine asthma

**DOI:** 10.1111/evj.14414

**Published:** 2024-09-14

**Authors:** Zsófia Nyerges‐Bohák, Levente Kovács, Ágnes Povázsai, Enikő Hamar, Péter Póti, Márta Ladányi

**Affiliations:** ^1^ Institute of Animal Sciences Hungarian University of Agriculture and Life Sciences Gödöllő Hungary; ^2^ Sportlógyógyász Kft Csengele Hungary; ^3^ Institute of Mathematics and Basis Science, Department of Applied Statistics Hungarian University of Agriculture and Life Sciences Gödöllő Hungary

**Keywords:** autonomic nervous system, equine asthma, heart rate variability, horse

## Abstract

**Background:**

Equine asthma in severe form (severe equine asthma [sEA]) shares remarkable similarities with human asthma. Human studies detected changes in the autonomic nervous system function in asthmatic patients based on heart rate variability (HRV) analysis.

**Study design:**

Observational study.

**Objectives:**

To investigate the relationship between sEA and HRV in horses.

**Methods:**

Twenty horses diagnosed with sEA and 20 asymptomatic (non‐sEA) horses were investigated. SEA horses showed clinical signs. The RR intervals of the ECG were recorded for 1 h at rest between 9 AM and 11 AM using a heart rate (HR) monitor. HRV data were calculated using Kubios software. Parameters recorded for the sEA and non‐sEA groups were compared using one‐way MANOVA model. The significance level was set at *α* = 0.05.

**Results:**

SD2 (mean 99.6 ± SD 25.3 vs. 42.5 ± 17.1), SDNN (82.7 ± 20.7 vs. 41.3 ± 14.3), TINN (398.1 ± 104.9 vs. 209.3 ± 71.9), SD2/SD1 ratio (1.7 ± 0.2 vs. 1.1 ± 0.3), Total power (4740.2 ± 1977.9 vs. 1503.0 ± 1179.3), LF (2415.3 ± 1072.4 vs. 707.4 ± 649.9), SD1 (60.9 ± 15.9 vs. 39.2 ± 14.1), RMSSD (86.0 ± 22.6 vs. 55.3 ± 19.8) and HF (1575.8 ± 902.5 vs. 578.1 ± 491.1) were lower in sEA horses compared with the non‐sEA horses (*p* < 0.01 for each variable). SD2, SDNN, TNN, the SD2/SD1 ratio and Total power showed the greatest discriminatory power in differentiating the sEA and non‐sEA groups.

**Main limitations:**

Small sample size.

**Conclusion:**

Our findings indicate that like humans, asthmatic horses show an overall reduction in autonomic control. A relative increase of the parasympathetic modulation of the heart was also observed. After further investigations, HRV measurement might be a non‐invasive approach to monitor autonomic nervous system responses of sEA horses.

## 总结

**背景:**

马哮喘(EA)严重影响约10%‐15%的马匹。马哮喘EA与人类哮喘非常相似。人类研究通过心率变异性(HRV)分析检测到哮喘患者自主神经系统功能的变化。

**研究设计:**

观察性研究。

**研究目标:**

本研究旨在探讨休闲骑乘温血马中，严重马哮喘(sEA)与心率变异性(HRV)之间的关系。

**方法:**

对20匹被诊断为sEA的马匹和20匹无症状(非sEA)马匹进行调研。调研期间，sEA马匹表现出临床症状且未接受治疗。使用心率(HR)监测仪ECG，在上午9点至11点之间，记录1小时的RR间歇期。使用Kubios软件计算心率变异性数据。根据单因素多元方差分析模型，比较sEA组和非sEA组的记录参数。显著性水平设为α=0.05。

**结果:**

方差分析显示，sEA马的SD2(mean 99.6±s.d. 25.3 vs. 42.5±17.1)、SDNN(82.7±20.7 vs. 41.3±14.3)、TINN(398.1±104.9 vs. 209.3±71.9)、SD2/SD1比值(1.7±0.2 vs. 1.1±0.3)、TF(4740.2±1977.9 vs. 1503.0±1179.3)、LF(2415.3±1072.4 vs. 707.4±649.9)、SD1(60.9±15.9 vs. 39.2±14.1)、RMSSD(86.0±22.6 vs. 55.3±19.8)和HF(1575.8±902.5 vs. 578.1±491.1)均低于非sEA马(各变量p<0.01)。其中SD2、SDNN、TNN、SD2/SD1比值和TF，在区分sEA和非sEA群体方面，显示出最大的鉴别能力。

**主要局限性:**

样本量较小。

**结论:**

本研究结果表明，与人类相似，哮喘马匹的自主神经系统功能整体下降。我们还观察到心脏的副交感调节相对增加。进一步研究后，心率变异性测量可能成为一种无创的监测方法，用于监测sEA马对不同疗法的自主神经系统反应。

## 1 INTRODUCTION

Equine asthma (EA) is a complex disorder, the severe form (sEA) is common^1^ and is associated with coughing, exercise intolerance and increased respiratory effort.^2^ Decreasing the exposure to allergens and irritants is the primary method for managing the condition^3^; however, medical treatment can be necessary. Not all forms or stages of human asthma necessarily share the same attributes as EA but there are similarities between the two diseases.^4^

Analysis of the short‐term fluctuations in the heart rate variability (HRV) describe variations of both the instantaneous heart rate and the consecutive RR intervals of the ECG and is a widely accepted and validated assessment method for the modulation of autonomic nervous system in livestock species, including horses.^5^ Human studies have found characteristic changes in the autonomic nervous system function in connection with several cardiopulmonary diseases.^6^, ^7^ Several studies have analysed the relationships between human asthma and HRV.^6^, ^8^, ^9^, ^10^, ^11^, ^12^, ^13^, ^14^, ^15^, ^16^, ^17^, ^18^, ^19^, ^20^ Asthmatic patients usually have reduced HRV, and increased parasympathetic tone^8^, ^9^; however, some studies observed increased sympathetic activity in asthmatic patients.^16^ Modified autonomic control of the heart may arise from the change in lung compliance resulting from the vascular remodelling that characterises the disease.^21^ In sEA horses, chronic airway obstruction related to airway remodelling persists even in disease remission,^22^ therefore, autonomic nervous system activity might also change in horses as a consequence of the disease. The aim of the present study was to investigate the possible relationships between sEA and HRV in horses.

## 2 MATERIALS AND METHODS

### 2.1 Horses

Twenty horses diagnosed with sEA and 20 non‐sEA horses were used. They were client owned pleasure horses, which never compete, and only perform light work a few times a week. The age of the animals ranged from 7 to 18 (mean ± SD; 11.9 ± 3.0) years in the non‐sEA group and from 9 to 18 (mean ± SD; 13.7 ± 2.8) in the sEA group. Eight of the sEA horses were mares, 10 geldings, and 2 stallions. Ten of the non‐sEA horses were mares, 7 geldings, and 3 stallions. Horses were kept in roughly the same housing conditions in Pest County (daytime sandy paddock, nighttime stabled) and fed a commercial horse diet (i.e., oats and wheat bran) twice daily (6.00 AM and 6.00 PM). Animals had ad libitum access to hay and water. The experiment was performed between 10 and 28 June 2023 between 9.00 AM and 11.00 AM.

Horses included in the non‐sEA group had no history of any respiratory signs or poor performance. Horses were subjected to a physical examination consisting of temperature, heart rate and respiratory rate measurement, and auscultation of the heart, lungs, and gastrointestinal tract. Non‐sEA horses had no clinical signs and the resting respiratory rate was lower than 12 breaths per minute.

In the sEA group, the history collected, physical and endoscopic examination was performed and tracheal wash (TW) and bronchoalveolar lavage (BALF) samples were collected. Bacterial and fungal culture was performed from TW, cytology was performed on both TW and BALF samples. The diagnosis was based on a history of chronic cough at rest or during exercise,^2^ resting respiratory rate higher than 20 breaths per minute, mucus accumulation in the trachea with a score of 3 or more,^23^ and BALF cytology values of >20% neutrophils.^2^ All sEA horses met all these criteria. Horses with any signs of infectious disease such as fever, lethargy or anorexia, positive bacterial culture of the TW, or degenerate neutrophils, bacteria or fungal hyphae detected during cytology of the TW or BALF sample were excluded. HRV measurements were performed within 2 weeks of clinical diagnosis. The sEA horses were showing clinical signs at the time of HRV measurements and none of the horses had received medical treatment within the last 2 weeks prior to the experiment.

### 2.2 HRV measurements

RR intervals were recorded for 1 h using a portable HR monitoring system (Polar H10 sensor and Polar V800 watch; Polar Electro Oy Inc) validated for horses for the acquisition of HR signals for HRV measurements.^24^, ^25^ The Polar belt was placed on the horse's chest, and the electrode sites were positioned around the heart. The V800 receiver was attached to the horse's halter for the duration of the HRV recording. Horses were allowed to move freely in their usual environment during data collection. Each tested horse was allowed to become accustomed to the belt for half an hour before a 1‐h RR interval recording began. Five‐minute time intervals were used for the HRV analysis during which horses were at rest. For HRV analysis, the Kubios HRV software version 2.0 (Biosignal Analysis and Medical Image Group, Department of Physics, University of Kuopio, Finland) was used. One 5‐min time window was selected from the RR tachogram for each horse fulfilling the following criteria: (i) the HR was stable, (ii) the horse stood still undisturbed by humans or other horses, (iii) the horse finished with feeding or walking at least 5 min before data recording, (iv) no second‐degree atrioventricular block occurred. Each 5‐min segment was corrected for aberrant beats and errors using the custom Kubios correction algorithm, and the percentage of artefacts or premature heartbeats was under 2%.

HRV analysis was performed by time domain, frequency domain and geometric methods.^7^ Besides mean RR and mean HR data, the square root of variance of RR intervals (SDNN), square root of the mean squared differences of successive RR intervals (RMSSD) and the proportion of consecutive beat‐to‐beat intervals that differ by more than 50 ms (PNN50) were the calculated time domain data.^7^ In frequency domain analysis, the power spectral density was estimated using the fast Fourier transformation algorithm, and the total power, the low frequency (LF), the high frequency (HF) components of HRV, and the LF/HF ratio were calculated.^7^ The power of two spectral components were: LF; 0.04–0.13 Hz and HF; 0.13–0.26 Hz.^7^ The sequence of NN intervals was also expressed by a geometric pattern; baseline width of the RR interval histogram (TINN), Poincaré plot standard deviation perpendicular the line of identity (SD1), Poincaré plot standard deviation along the line of identity (SD2) and SD2/SD1 ratio were also estimated.

### 2.3 Data analysis

All statistical analyses were performed in R environment (v.4.3.1 R Core Team, 2023) using mainly the packages ‘MVN’, ‘caret’, ‘ranger’ and ‘DFA.CANCOR’.^26^, ^27^, ^28^, ^29^, ^30^, ^31^ The significance level was set at *α* = 0.05. Values of the LF parameter were transformed by the sqrt‐function to ensure the normality assumption. The normality of the residuals was accepted by the absolute values of their skewness and kurtosis as they were below 1.0. The homogeneity of variances was accepted in all cases by Levene's test (*p* > 0.05). In case of significant overall test, follow‐up univariate ANOVA was performed with Bonferroni's correction. The sEA and non‐sEA groups were compared according to the 14 variables (mean RR, mean HR, SDNN, RMSSD, NN50, pNN50, TINN, LF, HF, Total Power, LF/HF ratio, SD1, SD2, SD2/SD1) using one‐way multivariate ANOVA model.

To test whether the HRV variables could be used to differentiate the non‐sEA and sEA groups, Random Forest (RF) and Linear Discriminant Analysis (LDA) models were also run. The data set was first split into train and test data (with ratio 3:1). The models were trained on the train data set and were tested on the test data set. The hyperparameters of the RF models were set to their optimised values based on tuning (‘number of trees’: ntree = 300, the number of randomly sampled predictors: mtry = 5). Variable importance values were calculated by the Gini method.^32^

## 3 RESULTS

Sex (*p* = 0.67) and age did not differ between sEA or non‐sEA groups (*p* = 0.06). The overall one‐way MANOVA result was significant (Wilk's lambda = 0.18, *p* < 0.001, observed power >0.99) and the follow‐up univariate ANOVA resulted in highly significant sEA effect. Eleven out of 14 variables were significantly lower in sEA horses compared with non‐sEA group: SD2, SDNN, TINN, SD2/SD1 ratio, Total power, LF, SD1, RMSSD, HF, pNN50 and NN50 (*p* < 0.01). The observed power was above 0.99 for SD2, SDNN, TINN, SD2/SD1 ratio, Total power, LF, SD1, RMSSD and HF, while for pNN50 and NN50, the observed power was 0.96 and 0.87, respectively. The presence of sEA did not affect the mean RR, mean HR and LF/HF ratio (*p* = 0.13; Table 1). The confidence intervals of the sEA or non‐sEA groups did not overlap.

**TABLE 1:** Mean, standard deviation, lower and upper 95% confidence interval endpoints (LCI and UCI), of HRV parameters recorded for sEA and non‐sEA horses.

HRV parameters	Experimental group	Mean	Std. dev	LCI95%	UCI95%
Mean RR	Non‐sEA	1522.3	183.7	1436.3	1608.3
	sEA	1484.4	266.5	1359.6	1609.1
Mean HR	Non‐sEA	40.1	4.8	37.8	42.3
	sEA	41.6	7.5	38.1	45.2
SDNN	Non‐sEA	**82.7** ***	20.7	73.0	92.3
	sEA	41.3	14.3	34.6	48.0
RMSSD	Non‐sEA	**86.0** ***	22.6	75.5	96.6
	sEA	55.3	19.8	45.9	64.5
NN50	Non‐sEA	**95.0** **	23.1	84.2	105.8
	sEA	63.5	38.2	45.6	81.3
pNN50	Non‐sEA	**47.3** **	10.8	42.2	52.4
	sEA	30.3	17.2	22.3	38.3
TINN	Non‐sEA	**398.1** ***	104.9	348.9	447.2
	sEA	209.3	71.9	175.6	242.9
LF	Non‐sEA	**2415.3** ***	1072.4	1913.4	2917.2
	sEA	707.4	649.9	403.2	1011.5
HF	Non‐sEA	**1575.8** ***	902.5	1153.4	1998.2
	sEA	578.1	491.1	348.3	807.9
Total Power	Non‐sEA	**4740.2** ***	1977.9	3814.5	5665.9
	sEA	1503.0	1179.3	951.1	2054.9
LF/HF ratio	Non‐sEA	1.7	0.6	1.4	2.0
	sEA	1.3	0.7	1.1	1.7
SD1	Non‐sEA	**60.9** ***	15.9	53.5	68.5
	sEA	39.2	14.1	32.6	45.8
SD2	Non‐sEA	**99.6** ***	25.3	87.7	111.4
	sEA	42.5	17.1	34.5	50.5
SD2/SD1	Non‐sEA	**1.7** ***	0.2	1.6	1.8
	sEA	1.1	0.3	1.0	1.3

*Note*: Significantly higher values observed in the non‐sEA group compared with the sEA group are highlighted in bold.

*** *p* < 0.001;

** *p* < 0.01.

**FIGURE 1:**
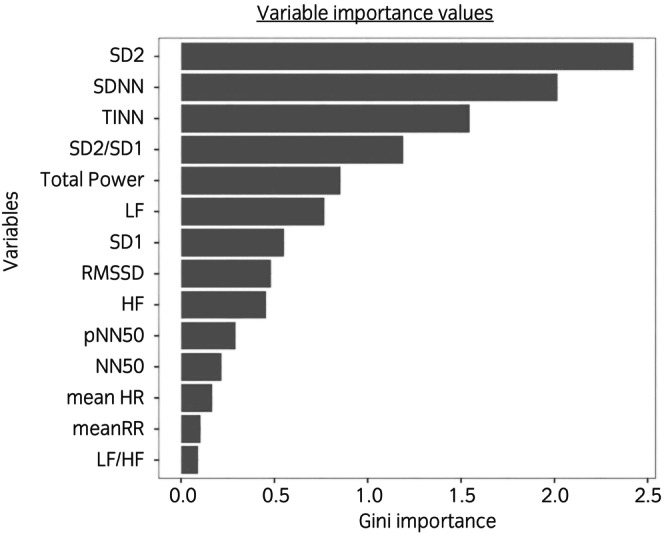
Variable importance values of the Random Forest model calculated by Gini method. It indicates the importance of each variable in the separation the severe equine asthma (sEA) and non‐sEA groups. The mean importance of the model was 0.80.

Both the RF and the LDA models confirmed that the variables with the greatest discriminatory power were SD2, SDNN, TNN, the SD2/SD1 ratio and Total power. In the RF model, the Gini importance indicated the relevance of each variable in differentiating the sEA and non‐sEA groups and the mean importance of the model was 0.80. These five variables exhibited Gini importance values that were higher than the mean importance. The models also indicated that LF, HF and SD1 were also essential in discriminating the two groups with their importance values not deviating notably from the mean importance with values higher than 0.50 (Figure 1). Variables RMSSD, NN50 and pNN50 exhibited slightly lower importance values (0.49, 0.36 and 0.19, respectively). The importance of mean HR, mean RR and the LF/HF ratio was negligible with their importances being below 0.14 (Figure 1).

In the LD model, the high (absolute) values of loadings (i.e., the correlations between each variable in the model and the discriminant functions) were seen in the same variables that were found significant by the MANOVA model: SD2 (0.63), SDNN (0.56), TINN (0.50), SD2/SD1 ratio (0.49), Total power (0.48), LF (0.51), SD1 (0.35), RMSSD (0.35) and HF (0.33) were significant lower in sEA horses. Variables pNN50 and NN50 were also decreased in sEA group with lower loadings (0.28 and 0.24, respectively). The loadings of mean HR, mean RR, and the LF/HF ratio were negligible (0.06, 0.04, 0.11, respectively) and these parameters differed not significantly between groups.

The Wilk's lambda of the discrimination function was 0.18 (*p* < 0.001), while the leave‐one‐out cross validation rate was 0.93. Both RF and LDA models' performances were similarly high (Table 2).

**TABLE 2:** The performance of the Random Forest (RF) and Linear Discriminant Analysis (LDA) models; accuracy, sensitivity, specificity, balanced accuracy together with the 95% confidence intervals (CI) calculated for the test set as well as the confusion matrices of the train and test data for the sEA and non‐sEA groups.

Statistics	RF model		LD model	
	Train set	Test set with CI	Train set	Test set
Accuracy	1.0	0.9 (0.71; 1.00)	1.0	0.8 (0.64; 0.97)
Sensitivity	1.0	0.8 (0.55; 1.00)	1.0	0.8 (0.55; 1.00)
Specificity	1.0	1.0 (0.93; 1.00)	1.0	0.8 (0.55; 1.00)
Balanced accuracy	1.0	0.9 (0.74; 1.00)	1.0	0.8 (0.55; 1.00)

		Prediction							
Confusion matrix		sEA	Non‐sEA	sEA	Non‐sEA	sEA	Non‐sEA	sEA	Non‐sEA
Reference	sEA	15	0	5	0	15	2	4	1
	Non‐sEA	0	15	1	4	0	15	1	4

## 4 DISCUSSION

In the present study, HRV parameters of horses with and without sEA were compared. Asymptomatic horses were healthy based on clinical history and physical examination; however, in this group BALF analysis was not performed and it is possible that animals with subclinical disease could have been included in the non‐sEA group. Both male and female individuals were included in both groups. Some sex‐related differences in HRV have already been reported in horses^33^, ^34^, ^35^; while other studies found no such differences in HRV.^36^, ^37^ It is unlikely that a sex effect would have influenced our results, especially since the asthmatic state was significantly independent of gender.

The results presented here indicate significantly lower values of SD2, SDNN, TINN, SD2/SD1 ratio, Total power, LF, SD1, RMSSD, HF, pNN50 and NN50 in sEA horses compared with non‐sEA horses. The RMSSD, PNN50, SD1 and HF reflect parasympathetic activity, while LF power is modulated by both sympathetic and parasympathetic outflows as well as by other factors, including baroreceptor activity.^12^ The Total power, SDNN, SD2 and TINN reflect overall autonomic activity, while the LF/HF power ratio and SD2/SD1 estimates the sympathovagal balance.^7^ Similar to our findings, a series of human studies observed reduced HRV, with a parasympathetic predominance in asthmatic patients,^6^, ^8^, ^9^, ^10^, ^11^, ^14^, ^15^, ^17^, ^19^, ^20^, ^38^, ^39^ which can manifest in the absolute increase of the HF,^6^, ^10^, ^11^, ^16^, ^20^, ^39^ or a decrease of LF with unchanged HF.^9^, ^38^, ^40^ Our main finding, that is, that the mentioned HRV indicators were reduced in sEA horses, indicates that the total autonomic activity of sEA horses is reduced. The autonomic nervous system exerts an important influence on the pathogenesis and control of asthma.^34^ Afferent pulmonary nerve pathways regulate cholinergic tone, which increases in parallel with the respiratory rate. Increase in cholinergic tone usually occurs in response to physiological and physiopathological stimuli.^3^, ^6^

In the present study, HF was lower in sEA horses compared with non‐sEA ones. In accordance with this finding, some human studies also reported a decreased power of the high frequency range in asthmatic patients.^8^, ^13^, ^14^ In these studies authors concluded abnormal autonomic nervous system modulation and disturbed cardiac vagal motor neuron activation.^13^, ^14^ However, these reports do not specifically discuss the reason for the lack of parasympathetic overload described elsewhere. A lesser decrease in HF than observed in LF parallel with a decreased SD2/SD1 and LF/HF ratio in sEA horses and suggest a slightly increased vagal overload associated with EA; however, total autonomic nervous system failure is a much clearer HRV observation in sEA horses. The vagal predominance observed here, may be the outcome of breathing against resistance which can result in swings in the intrathoracic pressure,^6^ causes fluctuation in cardiac performance and manifests in increased parasympathetic tone.^41^ Increased vagal tone is also considered to be pathogenetic component of bronchial hyperreactivity in human asthmatic patients.^41^ Airway hyper‐responsiveness to non‐specific agonists may also be present in horses diagnosed with heaves^42^, ^43^, ^44^, ^45^ and may be present even before any clinical signs are observed,^46^ and can also persist in asymptomatic horses.^47^ The association between hypersensitivity of asthmatic patients and a state of ‘vagotonia’ was first described in 1917 in humans.^48^ However, in our study, vagal hyperactivity was not found in sEA horses; the disruption of sympathovagal balance may be an important factor in mediating bronchial hyperreactivity in horses.

Based on our findings, it seems that vagal dysfunction is associated with severe asthmatic conditions, but its severity and the impact on the overall autonomic nervous system activity is unclear. Some studies found no evidence for a relationship between worsened HRV and a higher degree of disease in asthmatic human patients,^15^ whereas, similar to the findings presented here, others observed decreased sympathetic modulation in worse clinical condition in humans.^40^ Normally, when metabolic demand increases, the autonomic nervous system stimulates the lungs for enhancing the airflow, with a consequent improvement in gas exchange. This neural stimulus triggers a mechanical response following by the increase of the airways' diameter.^49^ This mechanism seems to be altered and ineffective in asthmatic patients. With exacerbation, the intrathoracic pressure increases and negatively affects the cardiac function of the horse.^50^ The changes in lung compliance and stretching of lung receptors might also have a role in reduced HRV as shown by SDNN and TINN parameters in humans.^8^ Controlled asthmatic patients can have normal autonomic nervous system function during rest,^16^ with a slightly lower sympathovagal response to orthostatic stimuli.^17^ Currently, the most likely assumption in human research is that the deterioration of the oxygen status is what directly depresses the sympathetic activity.^16^, ^39^, ^40^ In sEA, chronic airway inflammation also induces abnormal extracellular matrix deposition resulting in irreversible structural lung damage.^51^, ^52^ The consequent functional changes, like the increase in maximum transpulmonary pressure change, pulmonary resistance, and the decrease in dynamic lung compliance are almost entirely identical to the effects observed in humans.^53^ Therefore, it can be assumed that autonomic nervous system dysfunction experienced in horses in the present study could be associated with the same effects.

Severely asthmatic horses showed decreased SD1, SD2, SD2/SD1 ratio and TINN. SD2/SD1 showed the most important separation power between the groups. Dias de Carvalho et al.^54^ also demonstrated reduced SD1 and SD2/SD1 in humans with chronic obstructive pulmonary disease. SD2/SD1 corresponds to the standard deviation of the instantaneous variability of RR intervals^55^, ^56^ and indicates the parasympathetic influence on the sinoatrial node,^57^, ^58^ while SD2 is influenced by both parasympathetic and sympathetic pathways.^59^ Normally, horses have a relatively high vagal tone compared with other farmed species,^60^ and asthma exacerbation may have contributed to a relative parasympathetic hyperactivity. The reduction in SD2 and TINN in sEA horses suggests an overall reduction in the autonomic control, which was also supported by the time domain analysis. The SDNN and RMSSD indices were lower in sEA horses than in non‐sEA animals which is a common finding in uncontrolled asthmatic humans and suggest also a global autonomic damage caused by the disease.^6^, ^61^, ^62^

In conclusion, with a few exceptions, changes in HRV parameters in sEA horses are broadly similar to that of asthmatic humans. There is a decrease in the total HRV (SDNN, TINN, SD2, SD2/SD1 ratio, Total power) but an increase of the parasympathetic modulation of the heart was also detectable in sEA horses. In the present experiment, all of the asthmatic horses had sEA, and all were in the exacerbation phase of the disease. Since the degree of autonomic nervous system dysfunction and the severity of asthma may be related, the effects of mild equine asthma on HRV and the HRV pattern of exacerbation and remission phases remain to be investigated. HRV measurement has potential as a non‐invasive adjunct tool to monitor progression or response to therapy in sEA horses.

## FUNDING INFORMATION

Zsófia Nyerges‐Bohák was supported by the ÚNKP‐22‐4‐II New National Excellence Program of the Ministry for Culture and Innovation from the source of the National Research, Development and Innovation Fund. The study was supported by the National Research, Development and Innovation Office (Budapest, Hungary; Project ID: 2020‐1.1.2‐PIACI‐KFI‐2021‐00284).

## CONFLICT OF INTEREST STATEMENT

The authors declare no conflicts of interest.

## AUTHOR CONTRIBUTIONS

**Zsófia Nyerges‐Bohák:** Conceptualization; investigation; methodology; writing – original draft. **Levente Kovács:** Writing – review and editing; supervision; conceptualization. **Ágnes Povázsai:** Investigation; validation. **Enikő Hamar:** Investigation; formal analysis. **Péter Póti:** Investigation; writing – review and editing. **Márta Ladányi:** Software; data curation.

## DATA INTEGRITY STATEMENT

Dr. Zsófia Nyerges‐Bohák had full access to all the data in the study and takes responsibility for the integrity of the data and the accuracy of data analysis.

## ETHICAL ANIMAL RESEARCH

Approved by the Institutional Animal Welfare Committee of Hungarian University of Agricultural and Life Sciences Szent István Campus (MATE‐SZIC/836‐1/2023).

## INFORMED CONSENT

Not stated.

### PEER REVIEW

The peer review history for this article is available at https://www.webofscience.com/api/gateway/wos/peer-review/10.1111/evj.14414.

## Data Availability

The data that support the findings of this study are openly available in Harvard Dataverse at https://doi.org/10.7910/DVN/Y7BVQY.
